# The Book World of Medicine and Science

**Published:** 1907-08-31

**Authors:** 


### The Book World of Medicine and Science.

The Operations of General Practice. By Edward M. Corner and H. Irving Pinches. Oxford Medical Publications. (London: Henry Frowde, Hodder and Stoughton, 1907).

This must have been a difficult book to write. Its first readers, the publishers, have given their testimony by issuing it on good paper, which will stand much handling, and in a type which adds to the pleasure of reading it. The table of contents is well arranged, and the Index is copious and complete; about 170 excellent diagrams illustrate the text. The keynote of the book is contained in the chapter entitled "General" surgical cleanliness. This is the factor which is responsible for the admission of the general practitioner into the realm of general surgery. From first to last, in well-considered detail, this principle is kept in view, and once mastered the authors insist that it is not so much the question of operation as the question of policy which must determine a man's professional action. In proof of this there is much of common knowledge, much of information which most men might skim over were it not presented in such a happy form; but in this book no one will fail to be charmed by the side-lights which shine even upon common things. The criticism of common faults is neither irritating nor vexatious, and it deals admirably with the avoidable causes of failure. Mr. Arbuthnot Lane would be satisfied at the supersession of fingers by instruments—an evolution which marks the progress of aseptic surgery, and is more needful to the general practitioner than to the pure surgeon. The system by which knives may be sterilised by pure carbolic acid is not original, but is a valuable one to the man who lives far from his instrument-maker; by it the edge of the knife is not destroyed, and maintains its character as a cutting instrument. In the same association it would have been welcome to have had a short chapter on the making of incisions. It is not always the blunted knife which is responsible for the mincing incision, with serrated edges, which one sometimes sees, and which throws the general practitioner into contrast with the skilled operating surgeon. On the other hand, the caution never to use the handle of the knife as a dissector is clear and intelligible. A valuable chapter on anæsthetics precedes eleven chapters which deal with interference arranged under regional headings. The contents of these chapters describe operations of greater or less severity. The practitioner in selecting from among them such as are within his scope will be guided by his personal experience, for the book will appeal to varying classes of men, but no one, after reading the detail and cautions given, will have other than himself to blame if he unwisely ventures upon ground which is beyond his sphere. The authors have avoided all dictatorial character in their discussion of the subjects in the most marvellous way; they never transgress the bounds of fact and logical deduction. The chapter on the examination of the throat is specially good, but we miss the suggestion that, in adult patients, the soft palate may be perceptibly raised by causing the patient to focus his eyes on the face of the surgeon. Hernia is a subject of great interest to the general practitioner, and the method of dealing with the inguinal form is thoroughly described. But the call is often urgent, and when the practitioner has relieved a strangulation, the ligature and removal of the sac with a few stitches at the inguinal rings is a good termination for those who shrink from the major operation. Similarly, in dealing with varicose veins, it is suggested that Trendelenburg's operation is best combined with local removal of veins. The advantage claimed by Mr. Pearce Gould, when he introduced Trendelenburg's operation to English readers some five years since, was the small incision and the rapid recovery, but we must admit that when the veins are much enlarged they are better removed; while the ligature of the saphena gives a better result in the treatment of the small dark-coloured veins which lead to ulceration of the leg. We can find no fault in the remarks upon the treatment of stitch sinus as a "counsel of perfection"; but it lacks consideration from the standpoint of the general practitioner, who has sometimes to treat the condition left by the operation of another person. The patient frequently refuses, from the standpoint of expense, to go back to the operator, and with an altered anatomy re-opening the wound is not free from danger. It frequently suffices to plug the sinus with gauze in the form of a pyramid with its apex downwards, removing the gauze and syringing the sinus daily with a solution of phenol. When a few days have elapsed the stitch may be caught with a hooked probe, or, better still, with a small instrument figured some years since by Mr. Watson Cheyne. A cone of salicylic wool, left in for a day, will destroy the granulation layer of the sinus as deeply as it is septic, and healing immediately follows. Bier's treatment of septic inflammation of the limbs by passive congestion is favourably noticed, but we would rather advise it as a means of drenching the part with a home-made antitoxic serum, than as a matter of policy in the stage of weariness when "nothing is done." An after-incision is not a mark of failure any more than the swabbing of a throat after an injection of antidiphtheritic serum. The concluding chapter is occupied with preparation for operations by arrangement and in emergency. Perhaps too great a portion of it is a recapitulation of a preceding chapter on anæsthetics. This matter, as well as that on after-care, and the treatment of surgical shock would be more conveniently found in chapters I. and II. In preparation for emergencies most practitioners keep a bag, containing instruments frequently required, in such a state of purification as will permit of their being readily sterilised. Some indication of a formula of contents from the authors of this book would have been valuable. It is also convenient, in the rush of practice, to have a list of instruments useful in the more common operations; many men keep such lists, and add to, or curtail them as experience dictates. Here again the authors might have filled a page or two with useful hints.

The work is one for the consulting-room table of the busy practitioner. It will neither replace the larger text-book, nor the consultant, but it is a good index to both, and is certain of a hearty welcome from every earnest practitioner.

---

"Plaster of Paris, and How to Use It." By Martin W. Ware, M.D. (New York: Surgery Publishing Company. Pp. 88.)

Into this little treatise Dr. Ware has gathered much information on a subject which is apt to be neglected in the present day, when surgery is sometimes under the tyranny of the knife. Dr. Ware commences with a general consideration of the plaster-of-Paris bandage, mentioning the best kind of plaster to use and the best kind of bandage with which to use it; he also details the method by which the plaster and the bandage should be incorporated. He then describes the methods of applying the materials in the treatment of individual fractures, and concludes with concise accounts of the use of plaster of Paris in orthopædic surgery and in dental surgery. The descriptions are lucid and are accompanied by numerous and excellent illustrations.

